# Cloning & expression of SAK enzyme from *Staphylococcus aureus* in *E. coli* BL21-CodonPlus

**DOI:** 10.25122/jml-2021-0335

**Published:** 2022-06

**Authors:** Arafat Muttar, Intesar Tarik Numan

**Affiliations:** 1 Ministry of Higher Education and Scientific Research, Baghdad, Iraq; 2 Pharmacy Department, AL-Huda University College, Al Anbar, Iraq

**Keywords:** Staphylokinase (SAK), *E. coli*, 15 kDa, BL21-CodonPlus, Plasmid pET24b(+), SAK – Staphylokinase, r-SAK – recombinant staphylokinase, IPTG – isopropyl β-D-1-thiogalactopyranoside

## Abstract

Staphylokinase (SAK), also known as staphylococcal fibrinolysin, is a protein with a molecular mass of about 15 kDa produced by *Staphylococcus aureus*. Staphylokinase is synthesized in the late exponential phase, similar to streptokinase. The current study identified and predicted the protein SAK from *Staphylococcus aureus*. SAK is a fibrinolytic enzyme of the third generation that acts as an indirect activator of plasminogen. The current study cloned and expressed SAK protein isolated from *Staphylococcus aureus* and used in the form of a grid for enhancement of SAK Catalyst with PCR, disengagement, and change into articulation vector PET24b(+). The recombinant plasmid was changed into *E. coli* strain BL21 (codon additionally to 440) acceptance with isopropyl β-D-1-thiogalactopyranoside (IPTG).

## INTRODUCTION

Pathologies play a role in hemostasis failure and the development of thrombolytic agents. Staphylokinase (SAK) is a 136-amino corrosive (extracellular protein) that is produced in the late stages of a striking event [1–3]. The "agr" quality controller is clearly in charge of SAK. It causes plasminogen to form plasmin, which then digests fibrin clusters. The fibrin meshwork is disrupted as a result of this. Staphylokinase forms a 1:1 complex with plasminogen, revealing the dynamic site of the plasminogen particle [2]. This disrupts the fibrin meshwork, which can halt disease spread (thrombolytic) [4]. Staphylokinase was introduced into non-pathogenic *E. coli*, delivering recombinant staphylokinase (r-SAK) protein for thrombolysis. Recombinant SAK was thought to be more effective than streptokinase at breaking down platelet-rich vein thrombi. The treatment (staphylokinase) for these illnesses was greatly appreciated. As thrombolytic agents, staphylokinase, plasminogen activator, and urokinase were commonly used. Staphylokinase plays an important basic role in fibrinolysis. Staphylokinase activity was separated from methicillin-resistant *Staphylococcus aureus* (MRSA) strains in this study, and MRSA strains were isolated based on thrombolysis movement using a blood test from patients. This enormous quality was then introduced into *E. coli*, which produced recombinant staphylokinase (SAK) proteins against fibrinolysis.

## MATERIAL AND METHODS

We used bacteria from the Department of Molecular Biology, Faculty of Biology, Belarus State University, to clone recombinant plasmids (F 'proAB lacIqlacZM15 Tn10 (Tcr)/recA1 endA1 gyrA96 (Nalr) thi-1 hsdR17supE44 relA1 lac).

The PlacUV5 advertiser heavily influenced the quality of the bacteriophage T7 RNA polymerase In *E. coli* BL21 (DE3) cells (hsd, lady, cIts857, ind1, Sam7, nin5, lacUV5-T7gen1) lysogenic for the DE3 bacteriophage, and in *E. coli* BL21-CodonPlus (DE3)-RIPL cells containing enhanced duplicates of qualities rarely found in prokaryotes, t-RNA, and inducible articulation of the objective quality [5, 6]. As an expression vector, the plasmid pET24b(+) (Novagen, USA) was used. According to Mathew [7], total DNA was isolated. Ca+2 dependent transformation, DNA electrophoresis, and recombinant plasmid construction, isolation, and restriction analysis were all carried out in accordance with generally accepted experimental protocols. Fermentas enzymes and buffer systems were used (Lithuania).

Polymerase chain reaction (PCR) [8, 9] was performed in a standard composition mixture using a Veriti programmable thermostat. Bacteria were cultured in liquid media (LB). Bacteria were grown in 10–100 ml medium on a rocking chair at 180 rpm and 37°C. The optical density of the cells was measured spectrophotometrically every 30 minutes while plotting growth curves after dilution of the overnight culture and throughout the experiment. When OD600=1, IPTG was added to the experimental flasks at a concentration of 0.5 mmol/L for 4 hours of induction.

The Laemmli method was used to conduct an electrophoretic analysis of bacterial proteins in a 16 percent polyacrylamide gel (PAGE) under denaturing conditions with 0.1 percent SDS (sodium dodecyl sulfate) [10]. Before ligating the pET24b(+) vector, the gel was stained with a Coomassie blue R-250 solution. The sequence was then recloned into the expression vector pET24b(+) at the restriction enzymes Nde I and EcoRI sites. The resulting recombinant plasmid, designated PET-SAK, was used to transform *E. coli* strain BL21 cells (DE3).

Cells from clones that inherited the recombinant plasmid were grown in the presence of IPTG (isopropyl β-D-1-thiogalactopyranoside) to induce SAK enzyme expression. Centrifugation was used to pellet the cells, which were then mixed with a loading buffer and electrophoretically analyzed for cellular proteins.

### Preparation of pET24b(+) Vector Plasmid

A plasmid is a circular extrachromosomal DNA found in some microscopic organisms and yeast. The soluble lysis strategy was used in this trial to separate articulation plasmid pET24b(+) from an *E. coli* culture DH5 carrying the plasmid. Plasma extraction was conducted with the Qiagen kit, which is based on the alkaline lysis method and pET24b(+) vector digestion using extraction (Qiagen Kit). Staphylokinase synthetic DNA was synthesized by Integrated DNA Technologies (IDT) in California, USA, and SAK PCR product purification (Agilent Kit). Recombinant pET24b(+)-SAK plasmid preparation, colony PCR for selection of recombinants carrying the SAK gene Nde I, and EcoRI enzymes were used to digest the pET24b(+)-SAK vector. The pET24b(+)-SAK vector was used to transform competent DH5 alpha cells.

## RESULTS

**Figure 1:**
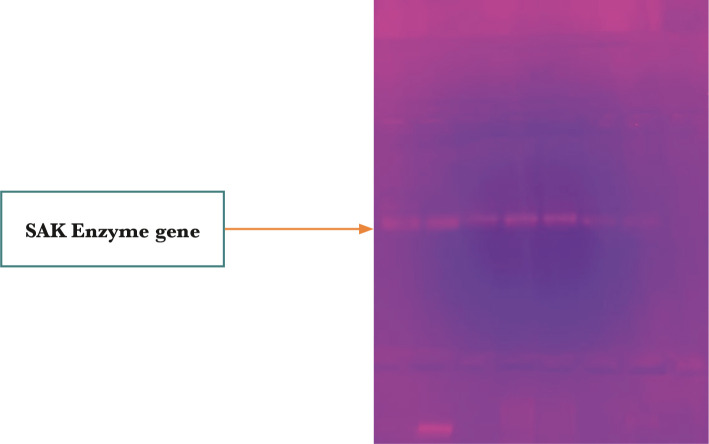
SAK Enzyme isolated from *Staphylococcus aureus* PCR in gel electrophoresis.

Figure 1 shows the SAK gene amplification using PCR-isolated enzyme from *Staphylococcus aureus*.

**Figure 2:**
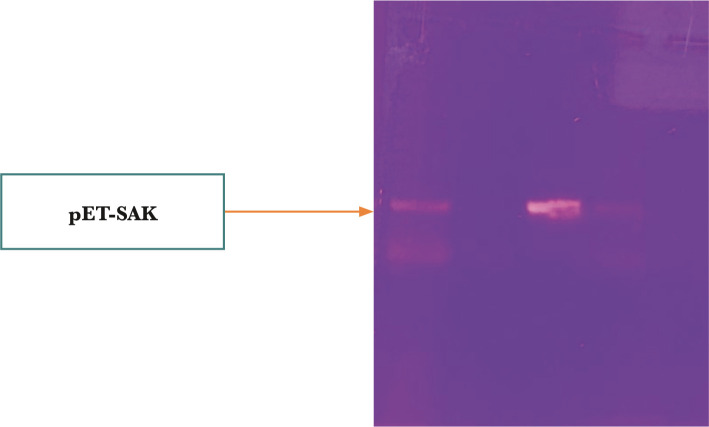
Transformation of expression host with recombinant plasmid (pET-SAK) in gel electrophoresis.

Then, *E. coli* microbes that produce a SAK chemical were created. Following this, the strain was replaced with a plasmid SAK. *E. coli* BL21 (DE3) clones that acquired the recombinant plasmid were filled in the presence of IPTG (isopropyl-D-thiogalactopyranoside) to activate quality articulation in SAK Catalyst. Changes in articulation have been made using a recombinant plasmid (pET-SAK) (Figure 2).

**Figure 3:**
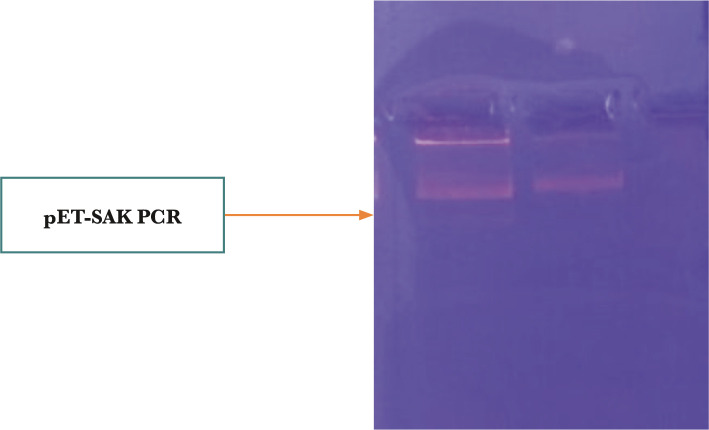
pET-SAK PCR in gel electrophoresis.

The enhancement item was implanted into the destinations for the limitations Nde I and Eco RI in a plasmid pET24b(+). Then the energy was put into sequencing, which revealed that the nucleotide sequence of the improved piece is equivalent to the full arrangement of the quality SAK Catalyst in the data set. The resulting recombinant plasmids are known as pET-SAK (Figure 3).

In addition to 440 (Jerpseth), which is lysogenic for bacteriophage DE3 and contains PlacUV5-advanced quality for bacteriophage T7 RNA polymerase, the type of *E. coli* BL21 Condon was changed. *E. coli* cells BL21 codon in addition to 440 were filled in the presence of IPTG for the acceptance of quality articulation in SAK protein.

**Figure 4:**
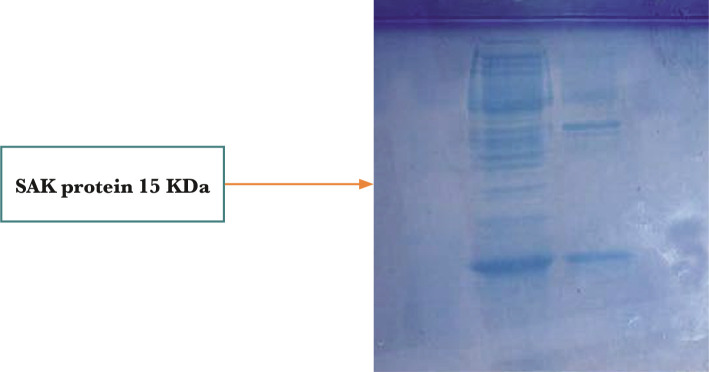
Purification protein and loading in SDS-page.

One possible explanation for the lack of or insufficient high-level expression of heterologous genes in bacterial cells is a difference in the frequency of occurrence of synonymous amino acid codons in prokaryotic cells (Figure 4).

## DISCUSSION

That is what the results show after enlistment with IPTG in bacterial cells containing the recombinant plasmid and collection of the protein relating to the sub-atomic load of a SAK chemical (15 kDa) [9, 10]. However, the level of protein accumulation was somewhat low for these types of articulation frameworks (11.3 percent of the absolute protein of cells as per computerized image analysis with TotalLab 2.0 (Nonlinear Elements Ltd., UK).

The fundamental pretended by bunch microscopic organisms, which is regarded as possibly the most important types of bacterium containing proteins and compounds, is the reason for the articulation and hereditary duplicate of SAK chemical. It is the coagulation of staphylokinase by recombinant microscopic organisms such as *E. coli*. The ability of *staphylococcus* to produce coagulase, which aids in blood clump arrangement, is possibly the most important aggregate used in the grouping of *staphylococcus*. Microfluongi, larynx, mosquitoes, and snakes are all used in some species [11]. Many of these proteins, known as recombinant staphylokinase proteins, have an effect on blood thickening. A few simple tests can distinguish *Staphylococcus* species from other high-impact and anaerobic electrocardiogram gram positives. *Staphylococcus aureus* is a facultative anaerobic (capable of becoming both vigorous and anaerobic) [12]. When bile salts are present, all species fill. Regardless of their role as *staphylococcus aureus*, they can cause a wide range of illnesses in humans and animals via poison production or entry. Several poisons are a common cause of food contamination because they can be produced by microscopic organisms in food stored incorrectly. *Staphylococcus aureus* is most commonly found in salivary organs, as are bacterial contaminations.

## CONCLUSION

The SAK standard was used. The SAK protein was 15 kDa in size and had high solvency in protein creation. The final stage was associated with the protein decontamination and detachment from the proteins delivered by *Escherichia coli* via a chromatography-based partition technique.
